# Supplementary material for: Content-rich biological network constructed by mining PubMed abstracts
Source: BMC Bioinformatics. 2004 Oct 8;5:147. doi: 10.1186/1471-2105-5-147 (PMC528731; doi:10.1186/1471-2105-5-147)
Supplement: Additional File 2 — The original results of the above study (non-essential files are deleted to keep the file size under the limit set by BMC bioinformatics). [file 1471-2105-5-147-S2.bz2 › chilibotAdditionalFile2/dip05/271ID2169352E1091/html/NR3C1_FOS.html]

 


 **NR3C1** and **FOS** 
  
Found 90 abstracts in PubMed, retrieved 05.  
 

 What does Google say? 
 PDF only 
| .edu only 

---

**Interactive relationship** (e.g. stimulation, inhibition, etc)

**Neutral relationship**- No group differences were found in plasma corticosterone levels or mRNA levels of glucocorticoid receptor  [ **NR3C1** ] , corticotropin releasing hormone, 5 HT 2C or c  **fos** .In conclusion, we have found that social interaction is a major component of the enriched environment regarding the effects on NGFI A and NGFI B expression.  Ref: 12809685 Neuroscience, 2003

**Non-interactive relationship** (e.g. studied together, co-existance, homology, etc.)

- This impairment may result from the absence of c  **Fos**  or glucocorticoid receptor  [ **NR3C1** ]  mediated transrepression.  Ref: 12766089 Invest Ophthalmol Vis Sci, 2003
